# A Community-Engaged Approach to Enhancing Participation in Genomic Research in Rare and Understudied Cancer Populations

**DOI:** 10.3390/ijerph22101468

**Published:** 2025-09-23

**Authors:** Saira Khan, Bailey Martin-Giacalone, Kayla Wallace, Briana Wilson, Christine Marx, Erin Linnenbringer, Jessica Mozersky, Melinda Bachini, Nancy Chollet, Dionne Stalling, Li Ding, Ryan C. Fields, Graham A. Colditz, Bettina F. Drake

**Affiliations:** 1 School of Medicine, Washington University in St. Louis, St. Louis, MO 63130, USA; b.martin-giacalone@wustl.edu (B.M.-G.); kaylawallace@wustl.edu (K.W.); briana.wilson@wustl.edu (B.W.); marxc@wustl.edu (C.M.); elinnen@wustl.edu (E.L.); jmozersky@wustl.edu (J.M.); lding@wustl.edu (L.D.); ryan_fields@urmc.rochester.edu (R.C.F.); colditzg@wustl.edu (G.A.C.); drakeb@wustl.edu (B.F.D.); 2 Participant Engagement Advisory Board, School of Medicine, Washington University in St. Louis, St. Louis, MO 63130, USA; melinda.bachini@cholangiocarcinoma.org (M.B.); nancy@finishingtouchstl.com (N.C.); dionnestalling@gmail.com (D.S.); 3 Cholangiocarcinoma Foundation, Herriman, UT 84096, USA; 4 Rare and Black, Clayton, MO 63105, USA; 5 University of Rochester Medical Center, University of Rochester, Rochester, NY 14627, USA

**Keywords:** community-engaged research, community-based participatory research, cancer, genomics

## Abstract

Few studies provide insights on how to incorporate community members’ perspectives of genomic research during the early phases of study development. Engaging with community members early and consistently throughout the research lifecycle could help identify and mitigate barriers to genomic research participation, particularly among groups with rare and understudied cancers. Methods: The Washington University Participant Engagement and Cancer Genome Sequencing (WU-PE-CGS) study formed a Participant Engagement Advisory Board (PEAB) consisting of patients, patient advocates, and patient advocacy organizations who represented the three understudied cancer populations: cholangiocarcinoma, early-onset colorectal cancer in Black Americans, and multiple myeloma in Black Americans. PEAB members were involved in PE-CGS from the time of the grant submission and provided input on key study procedures by participating in monthly project meetings and serving on the leadership team. PEAB recommendations are described in this process paper. Results: The PEAB provided key feedback on recruitment, consent, and survey development. Recruitment optimization focused on making the script more concise, tailoring to participant’s locale, and providing clearer participation expectations. Consent improvements prioritized key information, addressed data protection, and clarified the process of returning genetic results. Survey enhancements included refining scientific terminology and ensuring inclusivity across the cancer continuum. Conclusions: The PEAB provided valuable feedback that improved the development and implementation of WU-PE-CGS research processes. Incorporating the PEAB’s suggestions helped ensure that patients with rare and understudied cancers were successfully enrolled into the WU-PE-CGS. The PEAB will continue to contribute throughout all study phases.

## 1. Introduction

The underrepresentation of racially diverse participants in cancer genetics and genomics research is a longstanding issue. For example, 77% of the tumor samples in The Cancer Genome Atlas (TCGA) were from White participants, with significant underrepresentation of Asian and Hispanic participants compared to the relative proportion of the U.S. population [1]. The lack of diversity in genomic studies is also captured by the GWAS Diversity Monitor, a tool developed to track the total number of participants across all published genome-wide association studies (GWAS) [2]. As of October 2024, the tool showed that 94.5% of GWAS participants had genetic similarity to European ancestral groups [2]. The next largest participant group (3.9%) had genetic similarity to Asian groups, followed by participants with genetic similarity to African groups (0.6%). The stark overrepresentation of White participants and those most genetically similar to European ancestral groups means that understudied population groups may not be afforded the potential benefits of genomic research, including identification of genetic susceptibility variants, clinically actionable mutations, and targeted therapeutic opportunities [3]. This also limits scientific knowledge of the range of genetic variants and somatic mutations that may influence cancer development and survival.

Community engagement is a strategy to co-design studies and recruitment strategies that increase trust and promote opportunities for research participation [4]. These strategies comprise a continuum of research activities that promote shared priorities, mutual respect and trust, and shared decision-making among academic researchers and non-academic community members (hereafter termed “community members”). Community engagement is a well-established set of approaches necessary for considering ethical issues in genomics research, enhancing recruitment of underrepresented populations, and reducing health disparities [5]. The National Academies Roundtable on Genomics and Precision Health, the American Society of Human Genetics, in addition to ongoing genetic consortia including the Clinical Sequencing Evidence-Generating Research (CSER) and Electronic Medical Records and Genomic (eMERGE) network, call for community-engaged research approaches to make genomics research more equitable and increase participation of underrepresented groups [5,6,7,8,9,10,11,12]. They also recognize that community-engaged research can go beyond recruitment of underrepresented groups by incorporating community partners across several stages during and after the research study. However, as their reports allude to, few cancer genomics studies have yet to demonstrate how to use such community-engaged research processes to address these goals. Additionally, the literature on the use and success of community-engaged research processes in guiding large-scale cancer genomics research studies is limited.

The Cancer Moonshot Initiative was established in 2016 as a federal program to advance cancer research [13]. As part of the Moonshot Initiative’s goal to accomplish this through a focus on patient engagement, the Participant Engagement and Cancer Genomic Sequencing Network was funded to support five centers in this mission. The Washington University Participant Engagement and Cancer Genome Sequencing (WU-PE-CGS) is one of these centers and is focused on recruiting participants with cholangiocarcinoma, multiple myeloma among Black Americans, or early-onset colorectal cancer among Black Americans. Importantly, these cancers are rare or understudied and also reflect racial disparities in incidence and outcomes [14,15,16,17,18,19,20]. Additionally, the incidence of these three cancer types has increased over the past several decades. For multiple myeloma and early-onset colorectal cancer, incidence and mortality rates are significantly higher among African Americans than White Americans, and the reasons underlying these patterns are largely unknown [15,16,18,20]. To address these important knowledge gaps, the WU-PE-CGS formalized a community advisory board of non-academic community members to enhance study recruitment, consent, and survey design early in the research study design and data collection phases. This process paper provides a detailed description of the ways in which the WU-PE-CGS community advisory board and research team engaged in processes of research design and decision-making based on the community advisory board’s feedback. To date few studies have operationalized community engagement in cancer genomics specifically for rare cancer populations, and even fewer have documented their processes in detail.

## 2. Methods

### 2.1. Overview of the Participation Engagement Advisory Board (PEAB)

The WU-PE-CGS principal investigators established the Participant Engagement Advisory Board (PEAB) by emailing membership invitations to patients, patient advocates, and patient advocacy organizations who represented the three PE-CGS-focused populations—or those more broadly engaged in health disparities and rare diseases work. All invited individuals were required to be 18 years or older; some were a part of longstanding partnerships with WU-PE-CGS investigators, while others were newly invited for the purpose of the WU-PE-CGS. The composition of the PEAB was designed to represent all three cancer types included in this study (cholangiocarcinoma, multiple myeloma, and colorectal cancer). The PEAB initially comprised five members, and the number of members has fluctuated between five and eight members over the four years of the study. Three members withdrew because they were no longer a part of the partner organization or felt they no longer had time to participate.

The PEAB is a sub-unit within the Engagement Optimization Unit (EOU), a team focused on implementing and evaluating patient engagement approaches across the study. PEAB members were involved in the WU-PE-CGS at the time of grant submission, providing letters of support for the work of the investigative team. This study defined engagement with the PEAB as opportunities where PEAB members collaborated with the investigative team to provide input on PE-CGS processes, especially recruitment, informed consent, and survey development. Engagement primarily occurred through virtual PEAB monthly meetings beginning in April 2022 which are ongoing. PEAB members were also invited to engage in virtual Investigator Leadership meetings, which also occurred once per month, and addressed broader updates and challenges across the WU-PE-CGS units. PEAB members were compensated hourly for attending meetings and reviewing study materials.

### 2.2. PEAB Engagement Processes

Engagement with the PEAB followed principles of community-based participatory research (CBPR). An EOU member facilitated PEAB meetings in a semi-structured format and focused on engaging PEAB members in reviewing the following materials during different meetings: study recruitment phone script, study recruitment flyer, informed consent document, and participant baseline survey. The EOU team described the goal of the meeting and facilitated, in detail, discussion on the reasons to change or not to change aspects of the study process or documents. PEAB members provided verbal and/or written feedback on documents drafted by the investigative team. PEAB members were encouraged to use the chat function in Zoom and send written follow-up comments via email. EOU staff audio-recorded each meeting and documented feedback through written notetaking.

In a systematic-phased process, the EOU team edited materials based on PEAB suggestions, and shared the revised materials with the PEAB for additional feedback. Phase I: the PEAB were introduced to the study process of interest (e.g., recruitment, consent, or survey) and provided initial feedback and reactions; Phase II: the materials were designed and developed in conjunction with PEAB recommendations, existing literature, and IRB requirements; Phase III: the PEAB provided additional refinements to the developed study materials; Phase IV: the finalized materials were shared with the PEAB and implemented within the study. Any recommendations that could not be implemented were clearly explained by the study team along with the rationale for not implementing the change. EOU staff tracked the PEAB’s suggestions and any actions taken. For the baseline survey, the EOU drafted the survey, and then the PEAB members provided verbal feedback on the survey constructs during the meetings. The EOU then prepared the survey with the PEAB’s edits in REDCap (REDCap is a secured, web-based platform for hosting and managing online surveys and databases [21]), and each PEAB member took the survey and provided written feedback after each question/response option. For other printed materials (e.g., consent document, recruitment flyer) and the phone script, the PEAB provided feedback directly on electronic copies or verbally during the monthly PEAB meetings.

## 3. Results

### 3.1. Overview of PEAB Recommendations

The PEAB made key suggestions that enhanced study recruitment, informed consent, and survey development. A summary of the PEAB’s recommendations and the corresponding changes to informed consent, recruitment, and survey development are presented in Table 1, Table 2 and Table 3, respectively. Overall, the PEAB highlighted several potential barriers to study enrollment that the study team addressed including: (1) perception that the study team was asking for money, (2) belief that the researchers would exploit the use of tissues and/or specimens, (3) uncertainty about how study data and protected health information (PHI) would be used and shared, (4) confusion regarding what genetic results would be provided, and (5) concern about whether returned genetic test results would impact medical choices or healthcare.

### 3.2. Recruitment

The PEAB optimized the recruitment script by enhancing brevity, tailoring the script to the participant, and clarifying the expectations of participating in the study. For example, based on the PEAB’s recommendation, the study team tailored the recruitment script to the participant’s location by varying the script if the participant was local (St. Louis) or from outside the area. The PEAB also requested that during the recruitment process, study coordinators give more details about specimen collection and make it explicitly clear that participants’ specimens will not be exploited. As a result, the study team made changes to the recruitment script to emphasize that specimens would not be used for any purpose outside of the study and would not be shared with external organizations. The study team also clarified text to highlight that the study involves using existing samples and does not require participants to provide new samples over the five-year study period unless explicitly stated. The PEAB also provided suggestions to enhance the recruitment flyer. The initial recruitment flyer referenced the “Cancer Moonshot Initiative.” The PEAB felt that referring to this during recruitment may not be beneficial because participants may not be familiar with the initiative. Therefore, the flyer was adjusted to de-emphasize the “Cancer Moonshot” by placing in a less prominent position, and references to the “Cancer Moonshot” were supplemented with references to the National Cancer Institute to improve resonance and credibility. Moreover, the flyer title was changed from “Partner With Us” to “Help Us To Learn More” to better align with the intent of the study and make the call-to-action clearer and more engaging to potential participants.

### 3.3. Informed Consent

The PEAB enhanced the consent process by simplifying information, addressing data protection concerns, delineating the roles of the study team vs. participants’ healthcare providers, and clarifying the return of genetic results process. For example, based on PEAB feedback, the “Key Information” section of the informed consent document was shortened and re-ordered. Specifically, the EOU moved the “What will happen during this study?” sub-section to the beginning of “Key Information.” This helped ensure that participants received the most important information up-front. Additional feedback from the PEAB resulted in changes that more clearly explained that the study team will not pay for additional genetic testing and that study results will not impact on medical decisions or medical care provided by the participants’ healthcare providers.

### 3.4. Baseline Survey Development

The PEAB improved the baseline survey by suggesting that the EOU clearly defines genetic-related terms. As a result, the study team added specific definitions for genetic testing and genomic testing to the corresponding survey questions. The PEAB also pointed out that questions about “worry” also apply to patients in remission as well as patients with an active cancer diagnosis [22]. This resulted in the study team including this question for all participants. Finally, the PEAB highlighted many areas where clarity and brevity could be enhanced, resulting in changes such as removing the survey questions about how genetic tests would be returned and questions about collection of tissue samples.

### 3.5. PEAB Suggestions That Did Not Result in Changes to Recruitment, the Informed Consent Process, or Survey Design

The study team did not implement some PEAB suggestions in situations where the suggested changes were not feasible. For example, the PEAB suggested that the study team revise the text in the informed consent document from “you will not benefit from this study” to “you may or may not benefit from this study.” However, the Institutional Review Board (IRB) did not approve of this change. When providing feedback on the baseline survey, the PEAB reviewed the “Experiences with Racism” scale and recommended that the study team add “the time commitment of the doctor” as an option to capture this as a facet of racism. However, the study team opted not to make this change because the scale was previously validated [23]. Finally, the PEAB recommended that the recruitment script be tailored to each disease. The study team chose not to make this change due to the complexity of having three individual scripts for a single study.

## 4. Discussion

Community engagement is a foundational component of the PE-CGS. The overall goal of the PE-CGS is “to learn from—and work with—participants so we can help reduce health inequities in groups with rare and understudied cancers.” [24]. This goal also aligns with guidance from the American Society of Human Genetics [6]. Further, the relationship between the WU-PE-CGS and PEAB aligns with the principles of CBPR. CBPR is designed to bridge the gap between researchers and communities, leading to more culturally appropriate study designs and instruments and enrichment of data quantity and quality [25]. Within the WU-PE-CGS, the PEAB provided several insights and enhancements that would not have been identified by researchers alone. These insights and enhancements will ideally contribute to increased enrollment and participant engagement in the subsequent phases of the study. Whenever possible, the WU-PE-CGS deferred to PEAB recommendations.

To address increasing rates and disparities of the three selected cancers, the WU-PE-CGS aimed to increase research knowledge of the genomic landscape of tumors overrepresented in groups that have been underrepresented in cancer research [24]. However, many known barriers to participating in cancer genomics research exist: perceived costs, existing beliefs and knowledge, fears of discrimination based on results of genetic tests, privacy and confidentiality concerns, mistrust of the medical system, lack of information provided by providers, and confusion over various genetic tests and results [26,27]. These barriers can potentially impact Black Americans more than White Americans. As a result of these barriers, Black Americans may be less likely to use genetic testing and this can contribute to underrepresentation in cancer genomics research [1,27]. PEAB involvement early in the study design, from the time of grant development, was key in addressing these barriers. The specific and actionable concerns identified by the PEAB are well aligned with the known barriers to participation in research and genomic sequencing including mistrust of the medical system and fear of genetic discrimination. Notably, these concerns occurred early in the study process, at the time of recruitment and consent, and could have precluded participants from enrolling. Engaging with the PEAB early in the study design enabled the study team, in collaboration with the PEAB, to improve recruitment and consent processes before the start of the study.

In general, the feedback provided by the PEAB is consistent with broader recommendations for successful community-based research. A recurring theme of the feedback provided by the PEAB was the importance of presenting information in a concise manner, with the most important information first. This is consistent with previous findings that have shown shortened consent forms do not decrease comprehension of study procedures and can potentially increase participant engagement [28,29,30]. Moreover, the PEAB also highlighted the importance of tailored information and taking into account participant familiarity with federal initiatives. These recommendations are consistent with previous findings that highlight the importance of personalizing the informed consent process and developing resources that are appropriate for the community [3,6,31].

Ultimately, the WU-PE-CGS implemented the majority of suggestions recommended by the PEAB except in specific instances where the IRB did not approve, there was potential harm to data validity (changing a validated scale), or the change was both logistically complex and did not impact the key information being presented to participants (the key recruitment information was the same across all cancer types and thus the script was not tailored to cancer type). Throughout the project the study team had ongoing conversations with the IRB and shared PEAB feedback with the IRB. However, despite this in some instances PEAB recommendations could not be implemented due to regulatory requirements. In these instances, we honestly and clearly shared these decisions with the PEAB as soon as possible. We found that this clear and forthcoming communication strategy helped ensure that PEAB did not feel that their suggestions were being ignored. However, our experience highlights the tension between sometimes unflexible regulatory requirements and advisory board feedback. This demonstrates an ongoing need for new strategies that balance IRB requirements with community feedback in way that both maintains study integrity and ethical research processes while allowing for changes that respond to participant needs.

In the next study phase, the WU-PE-CGS will continue to work closely with the PEAB. Key next steps include returning genetic results to enrolled participants, administering the follow-up survey, and connecting participants with positive results to genetic counselors. The strong relationship formed between the study team and PEAB in the early stages of this study will help ensure continued collaboration throughout the implementation of the study. The PEAB members attended the Annual PE-CGS Meeting and indicated they would like to play a greater role in disseminating study findings as the study progresses.

Key strengths of our study include early PEAB involvement and the inclusion of three rare cancers that are increasing in incidence and disparities. Reversing these cancer trends will require that research, particularly genetic research, be representative of patients most likely to bear the burden of disease. Here, we have shown how early engagement of a community advisory board can help identify and address key barriers to participation in cancer genomic studies. However, it is important to note that the engagement approach used here requires ongoing resources making sustainability a challenge and PEAB members may not be representative of all patients with cholangiocarcinoma, early-onset colorectal cancer, and multiple myeloma.

Ultimately, the WU-PE-CGS will help address disparities among patients with cholangiocarcinoma, colorectal cancer, and multiple myeloma by increasing research knowledge of these rare and understudied cancers. Importantly, the lessons learned in the study can be applied to other conditions beyond the three cancers included here. Key lessons learned, including how community member feedback can enhance the clarity of consent and recruitment materials and help identify potential participant barriers early in the study, can be applied to genomic research for other rare cancers or chronic conditions. The development of institutional policies regarding advisory boards could address key issues including long-term sustainability beyond the period of grant funding and effective processes for balancing regulatory compliance while still allowing the agility needed implement advisory board suggestions.

## 5. Conclusions

The PEAB provided valuable feedback on recruitment, consent, and survey development. Key recommendations from the PEAB highlighted the importance of being concise, clearly laying out the expectations of participants if they choose to enroll, addressing data security concerns, prioritizing key information in the consent process, clarifying how genetic results will be returned, and plainly defining scientific methodology. Implementing the suggestions made by the PEAB will help ensure the success of subsequent phases of the WU-PE-CGS. Our findings highlight the importance of community engagement for genomic research, particularly for rare and understudied cancers.

## Figures and Tables

**Table 1:** Participation Engagement Advisory Board (PEAB) Recommendations for Recruitment in the Washington University Participant Engagement and Cancer Genome Sequencing (WU-PE-CGS) Study.

Optimization Suggested by PEAB Member(s)	Change Made by Study Team	Key Themes Identified
**Phone Recruitment Script**		
Recruitment script needs to be shortened Give minimum information to obtain consent Get right to point when calling, don’t spend a lot of time selling Washington University or asking for money Tell potential participants in the first 60 s that there’s a study regarding their cancer type	Revised the opening paragraph to immediately state the purpose of the call within the first 60 s, specifying that the study pertains to the patient’s cancer type. Reorganized content to prioritize critical information in the first few minutes of the call, enhancing engagement and efficiency. Ensured there was no language promoting Washington University or asking for financial contributions to prevent confusion or fear. Streamlined the script to focus on essential information, removing non-critical details.	Be concise Tailor information to participant
People need to know that their data helps them and other people with their cancer, but this could be shortened/said more simply	Reduced the length of the paragraph to avoid overwhelming the listener; focused on the key message about the importance of participation contribution to underserved communities and others with their cancer. Ensured that the revised wording is easy to understand and aligns with the overall goal of encouraging participation.	Be concise
Tailor recruitment script to participant locale	Adjusted the script to provide separate messaging for local and out-of-town participants, tailoring the information based on the participant’s location. Added a decision point in the script for callers to identify the participant’s area and select the appropriate statement.	Tailor information to participant
Make sure the participants know that their specimens/tissue and name won’t be exploited for other things and that the study does what the researchers say Don’t assume participants read consent form	Added a concise statement to the script reassuring participants that their specimens, tissue, and names will only be used for the stated purposes of the study and will not be shared with external organizations. Introduced a verbal confirmation step where participants were asked to repeat back key information about the study to ensure understanding.	Address distrust of research Ensure comprehension
Tailor script to each disease	This change was not made due to the complexity of having three individual recruitment scripts for a single study. In addition, key recruitment information was consistent and relevant for all cancer types.	Conflict with study procedures
Make sure it is clear to participants if more samples will be taken over the 5-year study-period	Updated wording to avoid potential misinterpretation, ensuring participants understand that the collection process is focused on already available tissue samples.	Enhance clarity
**Recruitment Flyer**		
Mentioned that highlighting the “Cancer Moonshot” initiative may not be helpful as participants may not be familiar with it	Adjusted the flyer to de-emphasize “Cancer Moonshot” by placing it in a less prominent position Supplemented references to “Cancer Moonshot” with a more universally recognized organization, the National Cancer Institute, to improve resonance and credibility.	Ensure comprehension
Recruitment Flyer Title ○ Title should be changed ○ Bile duct cancer should be mentioned sooner	Updated the flyer title from “Partner With Us” to “Help Us To Learn More” to better align with the intent of the study and make the call-to-action clearer and more engaging for potential participants. Ensured that the new title conveys a collaborative but approachable tone, emphasizing the value of participants’ contributions to the research. Adjusted the wording of the title to maintain clarity and relevance while prioritizing the specific cancer types being studied	Enhance clarity

Table 1 includes the optimizations suggested by the Participation Engagement Advisory Board (PEAB) regarding the recruitment process and the subsequent change made by the study team. Optimizations listed summarize direct quotes and feedback from PEAB members.

**Table 2:** Participation Engagement Advisory Board (PEAB) Recommendations for Informed Consent in the Washington University Participant Engagement and Cancer Genome Sequencing (WU-PE-CGS) Study.

Optimization Suggested by PEAB Member(s)	Change Made by Study Team	Key Themes Identified
**Informed Consent**		
Provide definitions of “genetic” and “genomic testing” as participants may use the terms differently	Updated all references to “genetic/genomic testing” to consistently use “genetic testing” throughout the consent process, aligning with common usage and simplifying the language. Although definitions were not included, the language was streamlined to ensure clarity and avoid confusion between genetic and genomic testing.	Ensure comprehension Enhance clarity
Enhance presentation of “Key Information” Make shorter Add consistency in order of diagnosis	Shortened the document, removing any unnecessary details to make the informed consent document more direct and easy to read Ensured that all mentions of different diagnoses appear in the same sequence, improving the flow and clarity of the consent information	Be concise Enhance clarity
Bring “What will happen during this study?” above “Key Information”	Moved the “What will happen during this study?” section to the beginning of the “Key Information” section for better flow and to ensure that participants receive the most important details upfront.	Enhance clarity Expectations of participants are clear up-front
Consider changing “you will not benefit from this study” to “you may or may not benefit from this study”	This change was not made as the IRB did not approve this change.	Conflict with institutional policy
Clarify who will have access to study data Make it clear that data will be shared beyond Washington University Specify that no Protected Health Information (PHI) will be shared	Added clarification to the “What will happen during this study?” section regarding data security, specifying that the data will be stored in secure computer databases and outlining who will have access to this information. Included a statement explaining that the study requires broad data sharing beyond the Washington University network, with potential for cross-collaboration between 4 and 5 institutions involved in the overall project. Added a clear assurance that no Protected Health Information (PHI) will be shared, emphasizing participant privacy and confidentiality.	Enhance clarity Address distrust of research
Add in examples of why genetic results may not be returned, and if that happens, what a person should expect	Added examples of why genetic testing results may not be returned, such as insufficient or inconclusive data. Included information on what participants can expect if results are not returned. Provided clear guidance to ensure participants understand the process and their options regarding participation in the event that results are not available.	Enhance clarity
Clarify that “the research team will not pay for further testing to confirm results for you or family members” applies to any genetic testing done during the course of research and does not apply to medical decision-making.	Revised the statement to clearly specify that “The research team will not pay for further testing to confirm results for you or family members” applies only to the results obtained through the research study. Added a clarification that this does not impact medical decision-making or any testing required for medical purposes, emphasizing that the research team’s financial responsibility is limited to the study itself. Ensured that the distinction between research-related results and medical care is clear to avoid confusion.	Enhance clarity Explain the role of the study team
Consider removing “if your results show X please follow up with your doctor”	Removed the statement “if your results show X, please follow up with your doctor”. Reworded the text to focus on the study’s role in providing research results while clarifying that any medical decisions or follow-up should be discussed with a healthcare provider, without implying direct medical guidance from the study.	Clarify role of study team vs. healthcare providers
Consider adding in secondary point of contact if genetic results cannot be returned	This change was not made due to IRB regulations	Conflict with institutional policies

Table 2 includes the optimizations suggested by the Participation Engagement Advisory Board (PEAB) regarding the consent process and the subsequent change made by the study team. Optimizations listed summarize direct quotes and feedback from PEAB members.

**Table 3:** Participation Engagement Advisory Board (PEAB) Recommendations for Survey Development in the Washington University Participant Engagement and Cancer Genome Sequencing (WU-PE-CGS) Study.

Optimization Suggested by PEAB Member(s)	Change Made by Study Team	Key Themes Identified
**Survey Development**		
Terms including tissue samples, genetic testing, and genomic testing may need clarifications	Removed the question regarding the collection of tissue samples, as it was unclear to participants. Decided to obtain this information at a later point in the study, ensuring it is clearer and less likely to cause confusion.	Enhance clarity
When asking participants if they want their results returned, the survey should include exactly what the results would consist of	Removed the question asking participants if they wanted results as it lacked specific details on what the results would consist of. Incorporated this information into the onboarding process, where participants can receive a full explanation of the various ways results may be returned to them.	Enhance clarity
Ask questions about “worry” to participants in remission as well since they have the same worries when it comes to recurrence	Expanded the cancer worry section to include individuals in remission Adjusted the wording of the section to ensure they are relevant and appropriate for both current cancer patients and those in remission.	Importance of patient perspectives
Add the time commitment of the doctor as an option in the “Experiences with Racism” scale	Not changed as this was a validated scale	Conflict with study procedures
Survey Testing ○ Identified typos ○ Identified questions with response errors ○ Suggested clarification of terms	Fixed typos and other errors as identified	Enhance clarity

Table 3 includes the optimizations suggested by the Participation Engagement Advisory Board (PEAB) regarding the survey and the subsequent change made by the study team. Optimizations listed summarize direct quotes and feedback from PEAB members.

## Data Availability

This is a process paper. There is no data to share beyond what is already presented in the manuscript.
